# Alveolar Type II Cell Damage and Nrf2-SOD1 Pathway Downregulation Are Involved in PM_2.5_-Induced Lung Injury in Rats

**DOI:** 10.3390/ijerph191912893

**Published:** 2022-10-08

**Authors:** Rui Niu, Jie Cheng, Jian Sun, Fan Li, Huanle Fang, Ronghui Lei, Zhenxing Shen, Hao Hu, Jianjun Li

**Affiliations:** 1 Medical College, Xi’an Peihua University, Xi’an 710061, China; 2 Department of Pharmacology, School of Basic Medical Sciences, Health Science Center, Xi’an Jiaotong University, Xi’an 710061, China; 3 Department of Environmental Science and Engineering, Xi’an Jiaotong University, Xi’an 710049, China; 4 Basic Medical Experiment Teaching Center, Health Science Center, Xi’an Jiaotong University, Xi’an 710061, China; 5 School of Public Health, Health Science Center, Xi’an Jiaotong University, Xi’an 710061, China; 6 Key Laboratory of Environment and Genes Related to Diseases, Xi’an Jiaotong University, Ministry of Education of China, Xi’an 710061, China; 7 Key Lab of Aerosol Chemistry & Physics, SKLLQG, Institute of Earth Environment, Chinese Academy of Sciences, Xi’an 710061, China

**Keywords:** PM_2.5_, PM_2.5_ constituents, lung injury, oxidative stress, alveolar type II cells

## Abstract

The general toxicity of fine particulate matter (PM_2.5_) has been intensively studied, but its pulmonary toxicities are still not fully understood. To investigate the changes of lung tissue after PM_2.5_ exposure and identify the potential mechanisms of pulmonary toxicity, PM_2.5_ samples were firstly collected and analyzed. Next, different doses of PM_2.5_ samples (5 mg/kg, 10 mg/kg, 20 mg/kg) were intratracheally instilled into rats to simulate lung inhalation of polluted air. After instillation for eight weeks, morphological alterations of the lung were examined, and the levels of oxidative stress were detected. The data indicated that the major contributors to PM_2.5_ mass were organic carbon, elemental carbon, sulfate, nitrate, and ammonium. Different concentrations of PM_2.5_ could trigger oxidative stress through increasing reactive oxygen species (ROS) and 8-hydroxy-2′-deoxyguanosine (8-OHdG) levels, and decreasing expression of antioxidant-related proteins (nuclear factor erythroid 2-related factor 2 (Nrf2), superoxide dismutase 1 (SOD1) and catalase). Histochemical staining and transmission electron microscopy displayed pulmonary inflammation, collagen deposition, mitochondrial swelling, and a decreasing number of multilamellar bodies in alveolar type II cells after PM_2.5_ exposure, which was related to PM_2.5_-induced oxidative stress. These results provide a basis for a better understanding of pulmonary impairment in response to PM_2.5_.

## 1. Introduction

Air pollution, typified by higher levels of fine particulate matter (PM_2.5_), has recently become a great concern for public health. Atmospheric PM_2.5_ refers to atmospheric particles with aerodynamic diameters less than or equal to 2.5 μm, which is a crucial risk factor to health and the environment around the world. Accumulated evidence has shown that PM_2.5_ can traverse the blood–air barrier of alveoli and deposit in the lung [1,2]. Epidemiological studies have indicated that long-term exposure to PM_2.5_ can cause extensive damage in the respiratory, cardiovascular, immune, and endocrine systems, even within hours to days of exposure in susceptible individuals [3,4,5]. Convincing evidence demonstrates that PM_2.5_ increases the risk of ischaemia-like injuries and induces developmental neurotoxicity and dysfunction of dopaminergic neurons [6,7,8]. Furthermore, atmospheric pollutants have their own health risk profile and have been associated with many diseases [9,10]. Apart from primary prevention, there is little effective treatment or clear targets for PM_2.5_-induced diseases.

Ambient PM_2.5_ has been linked to adverse health effects, mainly due to its chemical constituents. The composition of PM_2.5_ is complicated, including heavy metal elements, bacteria, viruses, inorganic ions, organic pollutants and other harmful substances. Moreover, components of PM_2.5_ vary from region to region and from source to source, which may greatly affect toxicity [11]. Understanding the local health effects of PM_2.5_ constituents is vital in making air pollution control policies from a public health perspective. PM_2.5_-containing organic matter and acidic ions are important in the regulation of oxidative stress and inflammation during haze episodes [12]. Previous research found significant positive associations of emergency room visits with organic carbon (OC) and elemental carbon (EC) ratios in PM_2.5_ mass [13]. PM_2.5_-induced oxidative stress has been considered as a vital mechanism of PM_2.5_-mediated toxicity [2]. Oxidative stress presents a critical imbalance between the production of reactive oxygen species (ROS) and antioxidant defenses. PM_2.5_-induced ROS directly interacts with antioxidant enzymes, such as superoxide dismutase (SOD), glutathione peroxidase and catalase, resulting in a loss of enzymatic activity [14].

Several PM_2.5_ toxicology studies have shown that PM_2.5_-induced lung injury is closely associated with inflammation and autophagy, and oxidative stress has been considered an important molecular mechanism [15,16,17]. However, the underlying mechanism of PM_2.5_-induced lung injury has not been fully explained. In the present study, PM_2.5_ samples were collected and examined in Xi’an, China, and the extent of PM_2.5_ damage to the lung and the potential mechanisms were assessed in PM_2.5_-exposed rats.

## 2. Materials and Methods

### 2.1. PM_2.5_ Sample Collection and Elution

Samples of PM_2.5_ were collected on the rooftop (~10 m above the ground) of a three-story building on the campus of the Institute of Earth Environment, Chinese Academy of Sciences, which is located in the urban center of Xi’an. PM_2.5_ were collected in prebaked (450 °C, 6–8 h) quartz fiber filters by a high-volume (1.13 m^3^/min) sampler (TISCH, Cleves, OH, USA), with an approximately 24 h sampling time each day from 5 December 2017 to 18 January 2018. After sampling PM_2.5_, the quartz filter membranes were eluted by an ultrasonic oscillator in methanol for 3 × 40 min and filtered through 8-layer sterile gauze. A freeze-drying technique was used to acquire dried samples, which were stored at −20 °C. The suspensions were swirled for 30 min before treatment in animals.

### 2.2. Animal Experiments

Adult male Sprague–Dawley rats weighing 200–220 g (6-week-old) were purchased from the Experimental Animal Center of Xi’an Jiaotong University (Xi’an, China). Rats were housed under standard conditions for one week before exposure. Thirty-two rats were separated into four groups (eight rats per group): control (0.9% saline), low-dose (5 mg/kg, PM_2.5_-L), medium-dose (10 mg/kg, PM_2.5_-M), and high-dose (20 mg/kg, PM_2.5_-H) PM_2.5_. After 3% isoflurane anesthesia, rats were intratracheally injected with 0.9% saline, 5 mg/kg PM_2.5_, 10 mg/kg PM_2.5_ or 20 mg/kg PM_2.5_ (1 mL/kg·body weight) suspensions every 4 days for eight weeks. All injections were performed between 08:00 and 09:00 a.m. to avoid circadian rhythm-induced variation. The suspensions were freshly prepared by diluting the stock solution with 0.9% saline. For seriously polluted Chinese cities, the reported PM_2.5_ concentrations were frequently above 250 μg/m^3^ lasting for 2 months (the data were obtained from China Air Quality Online Monitoring and Analysis Platform, www.aqistudy.cn, accessed on 31 August 2022). For adult rats weighing 250 g, the respiratory volume was 0.105 m^3^/day. Therefore, the representative inhaled PM_2.5_ amount was approximately 0.026 mg/day (0.105 m^3^/day × 250 μg/m^3^) for each rat. Given the interspecies uncertainty factor (10–100-fold) [18], the high dose used in this study was set as 20 mg/kg (0.026 mg/day × 4 day × 50-fold/0.25 kg), which was comparable to the ambient levels of PM_2.5_ in many Chinese cities. When the test was finished, all rats were euthanized with sodium pentobarbital (200 mg/kg) via an intraperitoneal injection.

### 2.3. Chemical Analysis of the PM_2.5_ Samples

Experimental procedures were carried out as described in our previous research [19]. The carbonaceous concentrations, including OC and EC, in PM_2.5_ samples were measured. A piece of the filter was extracted with Milli-Q water and filtered through a polytetrafluoroethylene syringe filter. Next, the water extract was assessed for water-soluble inorganic ions (Cl^−^, F^−^, NO_3_^−^, SO_4_^2−^, Na^+^, NH_4_^+^, K^+^, Mg^2+^, Ca^2+^) and for water-soluble organic carbon (WSOC).

### 2.4. 8-Hydroxy-2′-Deoxyguanosine (8-OHdG)

The lung tissues isolated from rats were centrifuged at 14,000 rpm for 15 min at 4 °C. Supernatants obtained by this procedure were utilized for the evaluation of the concentration of the oxidative stress parameter 8-OHdG according to the manufacturer’s instructions (Nanjing Jiancheng Bioengineering Institute, Nanjing, China).

### 2.5. Reactive Oxygen Species (ROS)

The level of ROS was determined by a 2,7-dichlorofluorescin diacetate (DCFH-DA) commercial kit according to the manufacturer’s instructions (Nanjing Jiancheng Bioengineering Institute, Nanjing, China) [20]. The cells from lung tissues were treated with DCFH-DA working solution and incubated for 30 min. After incubation, the culture medium was removed, and the cells were suspended in PBS. The precipitate was collected, and the fluorescence intensity of dichlorofluorescein (DCF) was monitored using a microplate fluorometer and luminometer (Thermo, Waltham, MA, USA) with an excitation wavelength of 500 nm and an emission wavelength of 530 nm. DCFH-DA itself has no fluorescence and can freely pass through the cell membrane. When DCFH-DA enters the cell, it is hydrolysed into dichlorofluorescin (DCFH) by the relevant enzymes in the cell. DCFH cannot penetrate the cell membrane, and therefore the probe can be easily contained within the cell. Given the presence of ROS, DCFH will be oxidized to DCF, an intense green fluorescent substance whose fluorescence intensity is proportional to the ROS level.

### 2.6. Transmission Electron Microscopy (TEM)

The lung tissue was minced into 1 mm^3^ cubes, fixed with 2.5% glutaraldehyde and 2% osmic acid, dehydrated and embedded in epoxy resin [21]. Ultrathin sections were collected onto 200-mesh copper grids, double stained with uranyl acetate and lead acetate, and then observed with a Hitachi H-7650 transmission electron microscope (Hitachi, Tokyo, Japan).

### 2.7. Haematoxylin–Eosin (HE) Staining

The histopathology of the lung was assessed by performing HE staining [22]. After the rats were sacrificed, the lung was quickly harvested on ice and fixed in 4% paraformaldehyde, dehydrated, cleared in xylene and embedded in paraffin. Six-mm-thick lung sections were prepared for HE staining and observed by microscopy (Olympus, Tokyo, Japan). For semiquantitative analysis, a total of 2 images from the lung in each rat were recorded (3 rats per group). Each image was scored for inflammation and macrophage accumulation in alveoli using a semiquantitative scale of 0 (no pathologic changes) to 4 (obvious pathology approaching maximal). Semiquantitative analysis of each lesion was scored by the presence of individual affected foci (score 1), increased frequency and size of individual lesions with occasional confluence (score 2), large areas of involvement and confluence (score 3), or complete lung involvement with the lesion in question (score 4) [23].

### 2.8. Masson’s Trichrome Staining

For Masson’s trichrome staining, 6-mm-thick lung sections were treated sequentially with haematoxylin and ferric oxide, acid fuchsin, phosphomolybdic acid, and acetic acid, and then the sections were mounted with neutral gum [24]. For semiquantitative analysis, a total of 2 images from the lung in each rat were recorded (3 rats per group). Images of the lung sections were obtained using a microscope (Olympus, Tokyo, Japan), and semiquantitative analysis of the immunohistochemistry staining was carried out with ImageJ software (NIH, Bethesda, MD, USA).

### 2.9. Enzyme-Linked Immunosorbent Assay (ELISA)

SOD activity (A001-1) and catalase activity (A007-1) in the lung tissue were determined using rat ELISA kits (Nanjing Jiancheng Bioengineering Institute, Nanjing, China). Experimental procedures were performed according to the manufacturer’s instructions.

### 2.10. Immunohistochemical Assay

For the immunohistochemical assay, 6-mm-thick lung sections were treated sequentially with an antibody against 8-OHdG (sc66036, 1:100, Santa Cruz, TX, USA), incubated at 37 °C for 1 h, kept in the refrigerator at 4 °C overnight, and then rinsed with phosphate-buffered saline (PBS). In addition, the samples were treated with biotinylated goat anti-rabbit secondary antibody solution (ready-to-use) and incubated at 37 °C for 30 min, followed by PBS washes. The samples were treated with newly prepared diaminobenzidine (Boster, Wuhan, China) staining solution for 2 min, followed by washing with PBS. The samples were restained with haematoxylin (Keygen, Nanjing, China) for 1 min, dehydrated, permeabilized, and mounted with neutral balata. Semiquantitative analysis of the immunohistochemistry staining was carried out with ImageJ software (NIH, Bethesda, MD, USA).

### 2.11. Western Blotting

Experimental protocols were carried out as described in our previous research [25]. Protein (20 μg) from lung tissues was separated by 10% SDS–PAGE and electrotransferred to polyvinylidene fluoride membranes. The membranes were blocked with 5% nonfat milk for 1 h at room temperature and incubated overnight at 4 °C with primary antibodies, including rabbit anti-nuclear factor erythroid 2-related factor 2 (Nrf2, CY5136, 1:1000, Abways, Shanghai, China), rabbit polyclonal anti-catalase (ABP0132, 1:1000, Abbkine, Wuhan, China), rabbit polyclonal anti-SOD1 (SAB32058, 1:1000, Signalway, MD, USA) and mouse monoclonal anti-GAPDH (AT0098, 1:5000, Engibody, DE, USA) antibodies. After washing three times with TBST, the membranes were incubated with corresponding horseradish peroxidase-conjugated secondary antibodies at room temperature for 2 h. The bands were visualized using the ECL system, and the band density was determined by ImageJ software (NIH, Bethesda, MD, USA).

### 2.12. Statistical Analysis

The data were expressed as the mean ± standard error of mean (mean ± SEM). Pearson’s correlation coefficient was used to examine correlations of constituents in PM_2.5_ with PM_2.5_ mass. Statistical analysis of multiple group comparisons was performed using Student’s t-test and one-way analysis of variance (ANOVA), followed by Bonferroni’s test. A value of *p* < 0.05 was defined statistically significant. All data were analyzed with the SPSS version 21.0 (IBM SPSS Statistics 21).

## 3. Results

### 3.1. Chemical Composition of Fine Particulates

**Figure 1:**
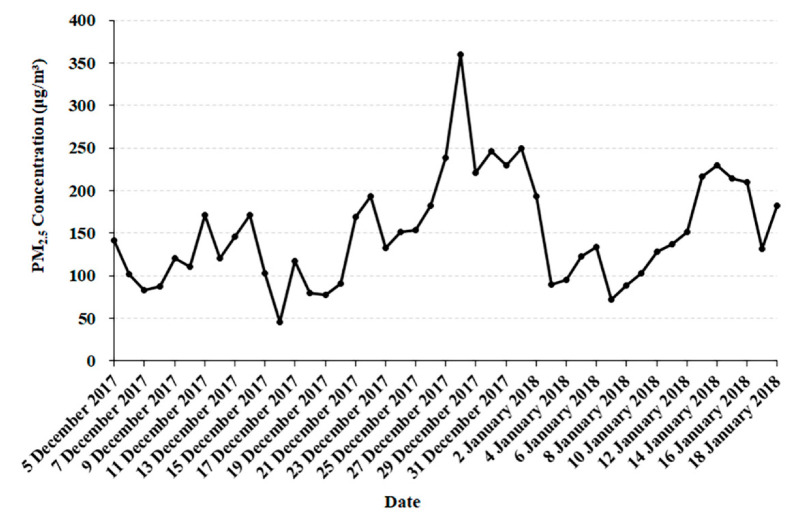
The daily concentrations of PM_2.5_ during collecting samples.

The mean daily average concentration of PM_2.5_ was 150.89 μg/m^3^ in Xi’an during the collection of PM_2.5_ samples (Figure 1 and Table 1), which was 10 times higher than the global guidelines set by the World Health Organization (annual average: 15 μg/m^3^). In addition, the average concentrations were 25.86 μg/m^3^ for OC and 5.05 μg/m^3^ for EC, accounting for 17.14% and 3.34% of the total PM_2.5_ mass, respectively (Table 1). In addition, the average WSOC concentration was 13.32 μg/m^3^, accounting for 8.83% of the total PM_2.5_ mass (Table 1). Other large contributors to PM_2.5_ mass were NO_3_^−^ (13.71%), SO_4_^2−^ (8.30%), NH_4_^+^ (7.00%), Cl^−^ (2.98%), and Ca^2+^ (2.02%). Generally, high correlations were observed for PM_2.5_ with OC (r = 0.82) and WSOC (r = 0.82) (Table 2). PM_2.5_ was weakly correlated with EC, SO_4_^2−^, NO_3_^−^, NH_4_^+^, K^+^, F^−^, Cl^−^, Mg^2+^, Na^+^ and Ca^2+^ (r = 0.55–0.77).

### 3.2. PM_2.5_ Induced Inflammation and Fibrosis in the Lung Tissue of Rats

**Figure 2:**
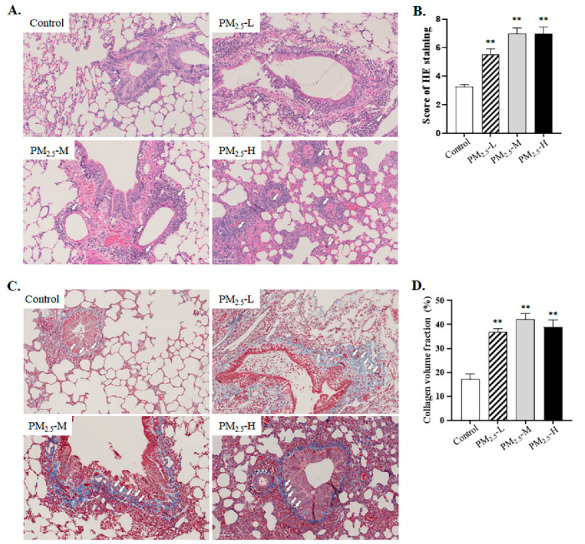
PM_2.5_ exposure induced inflammation and severe fibrosis in the lung of rats. (**A**) Pathological changes of lung tissues exposed to saline or different doses of PM_2.5_ for 8 weeks were detected by HE staining (magnification 200×, scale bar 100 μm). The white arrows indicted the lesions of inflammation. (**B**) Semiquantitative analysis of each lesion was scored for HE staining images. (**C**) Collagen deposition from saline- or PM_2.5_-treated rats was examined by Masson’s trichrome staining (magnification 200×, scale bar 100 μm). The white arrows indicted the lesions of collagen deposition marked by blue staining. (**D**) Collagen fibers were semi-quantified based on evaluation of Masson’s trichrome staining by the ImageJ software. The data were shown as mean ± SEM, ** *p* < 0.01 compared to the control group (*n* = 3 rats/group).

Morphological alterations of the lung in rats were evaluated by HE staining, as shown in Figure 2A. Lung structures were almost normal in the control group. Slight pulmonary inflammation was found in the low-dose PM_2.5_ exposure group, while pathological changes were increasingly noticeable with increased doses of PM_2.5_ (Figure 2A,B, Control vs. PM_2.5_-L, *p* = 0.002; Control vs. PM_2.5_-M, *p* < 0.001; Control vs. PM_2.5_-H, *p* < 0.001). To compare the extent of fibrosis in saline- and PM_2.5_-treated rats, collagen deposition was determined by Masson’s trichrome staining (Figure 2C). Masson’s trichrome staining appeared around blood vessels in the PM_2.5_ groups. As shown in Figure 2D, we found that the percentages of collagen volume fraction in lung tissues of PM_2.5_-treated rats were higher than those in saline-treated rats (Control vs. PM_2.5_-L, *p* < 0.001; Control vs. PM_2.5_-M, *p* < 0.001; Control vs. PM_2.5_-H, *p* < 0.001).

### 3.3. PM_2.5_ Induced Abnormal Ultrastructure in the Lung Tissue of Rats

**Figure 3:**
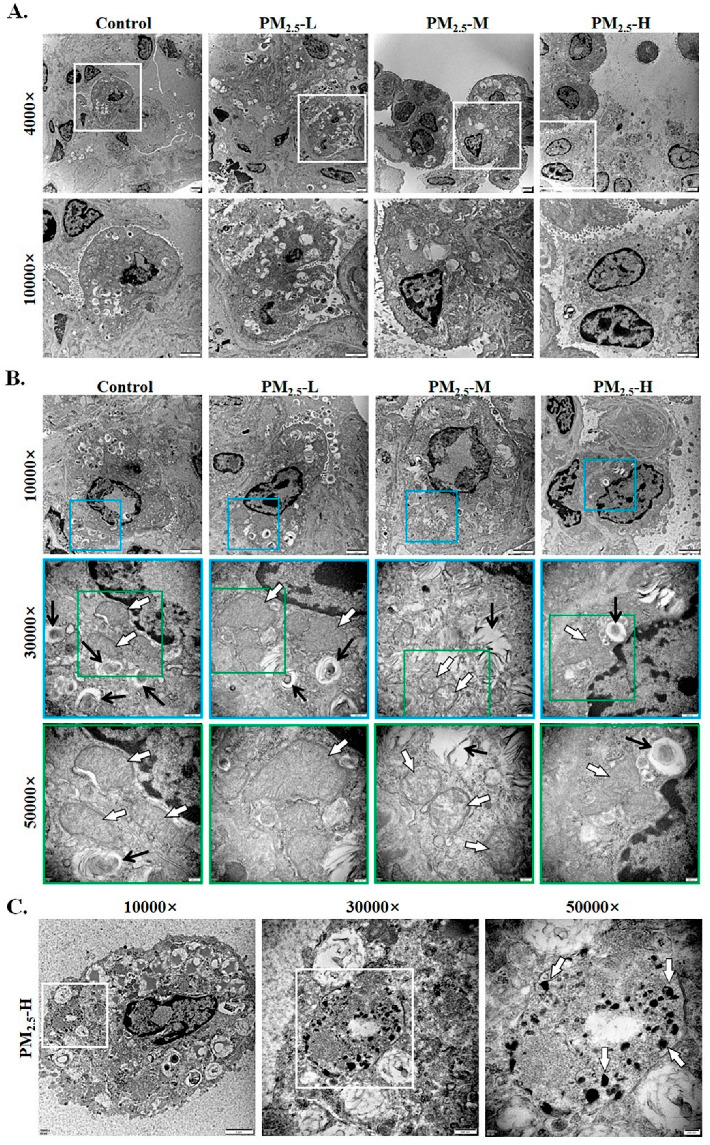
PM_2.5_ induced abnormal ultrastructure in the lung tissue of rats. (**A**) Changes of type II alveolar cells in the lung tissue of rats. The upper images were taken at 4000× magnification (scale bar 2 μm). The lower images were taken at 10,000× magnification (scale bar 2 μm). (**B**) Abnormal ultrastructure in the lung tissue of rats. Multilamellar bodies and mitochondria are shown with black arrows and white arrows, respectively. The upper images were taken at 10,000× magnification (scale bar 2 μm). The middle images with blue frames were taken at 30,000× magnification (scale bar 500 nm). The lower images with green frames were taken at 50,000× magnification (scale bar 200 nm). (**C**) PM_2.5_ deposition in the high-dose PM_2.5_ group. White arrows indicate PM_2.5_ (scale bar 200 nm).

The ultrastructure of the rat lung is shown in Figure 3. Alveolar type II cells can be identified by the presence of specific intracellular structures such as lamellar body producing surfactant [26]. Normal lung epithelial cells (alveolar type II cells) and multilamellar bodies were observed in rats in the control group. In addition, the TEM results of control rats revealed mitochondria with intact inner and outer membranes and well-ordered organelle morphology. However, under PM_2.5_ exposure, type II alveolar cells were obviously aggregated in the PM_2.5_ groups (Figure 3A). In addition, mitochondrial swelling and vacuolation with less defined cristae in the PM_2.5_ groups (Figure 3B) were found. Furthermore, PM_2.5_-exposed rats also displayed several structural changes, including excessive hypertrophic multilamellar bodies and alveolar collapse. Similar pathological features in alveolar type II cells represented in the PM_2.5_-L group were also found in the medium-dose and high-dose PM_2.5_ groups. The decreasing number of multilamellar bodies and the degree of mitochondrial swelling and vacuoles were found to be more visible with increased doses of PM_2.5_. Notably, obvious deposition of PM_2.5_ was observed only in the PM_2.5_-H group (Figure 3C).

### 3.4. PM_2.5_ Induced Oxidative Stress in the Lung Tissue of Rats

**Figure 4:**
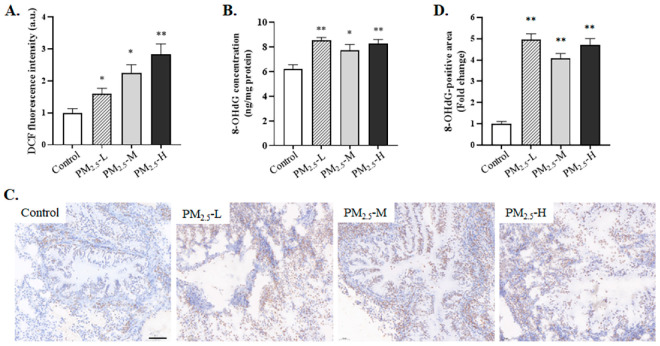
PM_2.5_ induced oxidative damage in the lung tissue of rats. (**A**) ROS level (*n* = 3 rats/group). The fluorescence intensity of DCF is proportional to the ROS level. (**B**) 8-OHdG concentration (*n* = 4 rats/group). (**C**) The representative immunohistochemical staining images of 8-OHdG expression in each group (scale bar 100 μm). (**D**) Semi-quantitative analysis of immunohistochemistry of 8-OHdG expression (*n* = 3 rats/group). The data are shown as mean ± SEM, * *p* < 0.05, ** *p* < 0.01 compared to the control group.

To assess whether PM_2.5_ exposure induced oxidative stress, the expression levels of ROS and 8-OHdG in the lung tissue were measured (Figure 4). The results showed that the ROS level in rats exposed to PM_2.5_ was significantly increased compared with that in control rats (Figure 4A, Control vs. PM_2.5_-L, *p* = 0.049; Control vs. PM_2.5_-M, *p* = 0.012; Control vs. PM_2.5_-H, *p* = 0.007). 8-OHdG is the most commonly used biomarker in oxidative damage. The concentration of 8-OHdG in lung tissue increased significantly in the different PM_2.5_ dose groups compared with the control group (Figure 4B, Control vs. PM_2.5_-L, *p* = 0.001; Control vs. PM_2.5_-M, *p* = 0.043; Control vs. PM_2.5_-H, *p* = 0.003). On the other hand, in the immunohistochemical assay, the 8-OHdG-positive area was obviously increased after rats were exposed to different doses of PM_2.5_ compared to control rats (Figure 4C,D Control vs. PM_2.5_-L, *p* < 0.001; Control vs. PM_2.5_-M, *p* < 0.001; Control vs. PM_2.5_-H, *p* < 0.001).

### 3.5. PM_2.5_ Decreased the Expression of Nrf2-Related Proteins in the Lung Tissue of Rats

**Figure 5:**
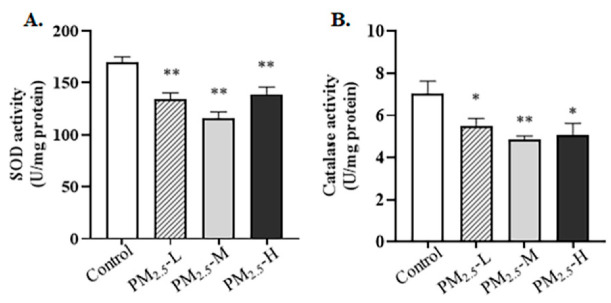
Changes of antioxidase activity in the lung tissue of rats exposed to PM_2.5_. (**A**) SOD activity; (**B**) catalase activity. The data are shown as mean ± SEM, * *p* < 0.05, ** *p* < 0.01 compared to the control group (*n* = 6 rats/group).

**Figure 6:**
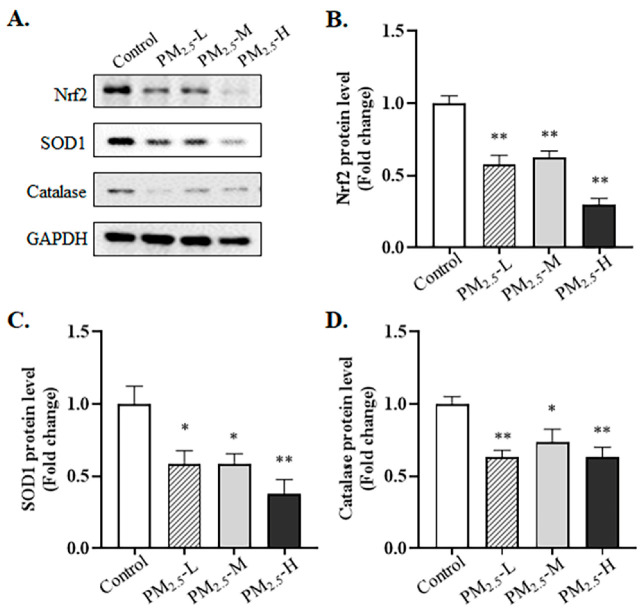
Changes of the Nrf2 signal pathways in the lung tissue of rats exposed to PM_2.5_. (**A**) The representative protein bands; (**B**) changes of the Nrf2 protein; (**C**) changes of the SOD1 protein. (**D**) changes of the catalase protein. The data are shown as mean ± SEM, * *p* < 0.05, ** *p* < 0.01 compared to the control group (*n* = 3 rats/group).

In addition, whether PM_2.5_ stimulated oxidative stress and perturbed the expression of the Nrf2 pathway needed to be determined. First, ELISA results indicated that PM_2.5_ exposure weakened antioxidase activity, including SOD and catalase activity, compared to the control group (Figure 5A,B, SOD activity: Control vs. PM_2.5_-L, *p* = 0.001; Control vs. PM_2.5_-M, *p* < 0.001; Control vs. PM_2.5_-H, *p* = 0.005; catalase activity: Control vs. PM_2.5_-L, *p* = 0.048; Control vs. PM_2.5_-M, *p* < 0.005; Control vs. PM_2.5_-H, *p* = 0.035). As shown in Figure 6A,B, Western blotting analysis suggested that the Nrf2 protein was obviously decreased after exposure to different doses of PM_2.5_ (Control vs. PM_2.5_-L, *p* = 0.006; Control vs. PM_2.5_-M, *p* = 0.005; Control vs. PM_2.5_-H, *p* < 0.001). On the other hand, rats in the PM_2.5_ groups had lower levels of the SOD1 and catalase proteins than the control group (Figure 6A,C,D, SOD1 protein: Control vs. PM_2.5_-L, *p* = 0.025; Control vs. PM_2.5_-M, *p* = 0.020; Control vs. PM_2.5_-H, *p* = 0.008; catalase protein: Control vs. PM_2.5_-L, *p* = 0.009; Control vs. PM_2.5_-M, *p* = 0.040; Control vs. PM_2.5_-H, *p* = 0.009).

## 4. Discussion

Given the spatial and temporal variability of PM_2.5_ physicochemical properties and toxicity, the aim of this study was to estimate the toxicological effects and underlying mechanisms of PM_2.5_ collected in the winter, the most polluted season, from Xi’an, one of the most heavily populated cities in China. The major contributors to PM_2.5_ mass were confirmed to contain OC, EC, sulfate, nitrate, and ammonium.

Recent studies have suggested that particle composition plays an important role in particle-associated adverse health effects [3,6,27]. The extractable organic compounds (a variety of chemicals with mutagenic and cytotoxic properties) contribute to various mechanisms of cytotoxicity [28,29]. In addition, the water-soluble fraction (mainly transition metals with redox potential) plays an important role in the initiation of oxidative DNA damage and membrane lipid peroxidation [30,31,32]. The OC/EC ratio is usually used to infer the emission sources of carbonaceous aerosols [33]. In the present study, the OC/EC ratios (average: 5.3) of PM_2.5_ samples were relatively high, suggesting the dominance of biomass and biofuel emissions compared to lower OC/EC ratios (range: 1.0–4.0) from fossil fuel (coal and vehicular exhausts) emissions.

The mammalian lung has a tree-like airway compartment that allows air transport, and honeycomb-like structures named alveoli, which are the functional units of blood–gas exchange. Thus, the alveoli are constantly exposed to outside environments and frequently encounter pathogens, particles and other harmful substances [26]. Alveolar type II cells, one of two types alveolar epithelium, play essential roles such as keeping the surface tension of alveoli and modulating lung immune responses [34]. In this study, histological examination of lung tissues from PM_2.5_-exposed rats showed a significant increase in inflammatory infiltration and cellular thickening of the alveolar walls with a strong tendency to fibrosis. In addition, the risk of fibrosing alveolitis was heightened in rats subjected to long-term PM_2.5_ inhalation. TEM analysis of lung tissue sections further confirmed that the impaired ultrastructure of alveolar type II cells contained mitochondrial destruction and abnormal lamellar bodies in the lungs of rats exposed to PM_2.5_. PM_2.5_ deposition was observed in rats, which indirectly suggested that severe haze PM_2.5_ events adversely affected pulmonary function. Furthermore, chronic exposure to PM_2.5_ decreased the production of mitochondrial antioxidants, which might also lead to increased susceptibility to oxidative mitochondrial injury [35]. Taken together, these changes provided evidence that the protective role of alveolar type II cells in the lung was attenuated and then led to lung injury in PM_2.5_-exposed rats.

It can be speculated that the above pathological changes in alveolar type II cells might be caused by the oxidative imbalance induced by PM_2.5_. Ho et al. confirmed that PM_2.5_-containing OC and acidic ions were vital in affecting oxidative stress and inflammation during haze episodes [12]. The oxidative stress mediated by PM might arise from mixed sources, including the direct generation of ROS from the surface of particles, soluble compounds such as transition metals or organic compounds and altered function of mitochondria generating ROS [36,37]. The production of ROS has been argued to play an important role in the primary cytotoxic effects of respirable exhaust particles and urban street PM. Excessive generation of ROS could overwhelm the antioxidant defense system and oxidize cellular biomolecules, such as disrupting the expression of antioxidant proteins and increasing levels of oxidative products. With an increase in PM_2.5_ dose, the level of ROS in rat lung tissue also increased. The antioxidant activity of SOD and catalase were significantly reduced. Moreover, the results of 8-OHdG, a marker of DNA damage, further indicated that PM_2.5_ induced oxidative stress.

Oxidative stress is crucial in the pathogenesis of PM_2.5_-induced lung damage. In response to oxidative stress, cells have different defense systems to regulate oxidative stress-derived damage [38]. Increasing evidence suggests that the oxidative stress-related Nrf2 pathway plays an important role in lung injury [39,40]. The transcription factor Nrf2 induced the expression of a great number of cytoprotective and detoxifying genes, and the Nrf2 pathway is an intrinsic mechanism of defense against oxidative stress [41]. In the present study, it is clear to observe that PM_2.5_, even at low dosage, could disrupt Nrf2 protein expression and its downstream related antioxidant proteins, such as SOD1 and catalase. These results demonstrated that the role of the antioxidant defense system regulated by Nrf2 was critical in PM_2.5_-induced lung tissue oxidative stress.

However, there were still many limitations in this study. First, intratracheal instillation has been widely applied in PM_2.5_ toxicological research, but real-world PM_2.5_ exposure is the best model for simulating humans breathing polluted air. Since the oxidative state is a dynamic process, different activation pathways may happen in long-term exposure. Future research could consider using a real-world PM_2.5_ exposure model to confirm and validate the results from the present study. Second, the present study did not determine the relationship between the chemical components of PM_2.5_ and the observed changes in pulmonary toxicity. Third, it was preferable to determine the linear and quadratic variable response to the increasing levels of PM_2.5_.

## 5. Conclusions

**Figure 7:**
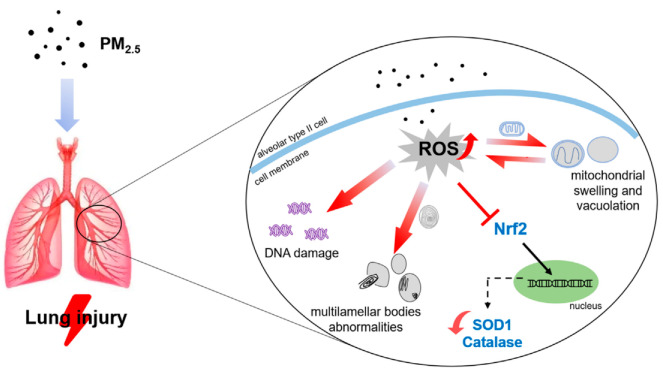
Possible pathways of PM_2.5_ exposure induced lung injury in rats.

In conclusion, our study demonstrated that low to high doses of PM_2.5_ may exacerbate pulmonary fibrosis and impair alveolar type II cells by increasing ROS level and lowering antioxidant proteins associated with the Nrf2 pathway, thereby inducing rat pulmonary toxicity (Figure 7).

## Figures and Tables

**Table 1:** Descriptive statistics for air pollutants in Xi’an, China.

Contents	Range (μg/m^3^)	Mean ± SEM (μg/m^3^)	Percent PM_2.5_ Mass (%)
PM_2.5_	45.00–360.03	150.89 ± 9.20	
OC/EC	2.74–9.24	5.30 ± 0.19	
OC	9.96–46.49	25.86 ± 1.36	17.14
WSOC	5.49–24.47	13.32 ± 0.78	8.83
EC	1.73–10.48	5.05 ± 0.27	3.34
Ca^2+^	0.22–8.90	3.05 ± 0.27	2.02
K^+^	0.32–5.35	1.48 ± 0.13	0.98
Na^+^	0.30–4.65	1.10 ± 0.13	0.73
Mg^2+^	0.03–0.47	0.17 ± 0.01	0.11
NH_4_^+^	2.45–30.64	10.56 ± 1.03	7.00
F^−^	0.01–0.27	0.11 ± 0.01	0.07
Cl^−^	1.19–14.46	4.49 ± 0.38	2.98
NO_3_^−^	4.45–62.44	20.69 ± 2.15	13.71
SO_4_^2−^	3.08–34.58	12.52 ± 1.19	8.30

Notes: The number of PM_2.5_ samples was 45. PM_2.5_, fine particulate matter; OC, organic carbon; EC, elemental carbon; WSOC, water soluble organic carbon.

**Table 2:** Correlations among PM_2.5_ mass and constituents in Xi’an, China.

	PM_2.5_	OC	WSOC	EC	Ca^2+^	K^+^	Na^+^	Mg^2+^	NH_4_^+^	F^−^	Cl^−^	NO_3_^−^	SO_4_^−^
PM_2.5_	1	0.82	0.82	0.55	0.56	0.68	0.73	0.77	0.59	0.62	0.74	0.65	0.55
OC		1	0.81	0.84	0.38	0.81	0.58	0.63	0.50	0.69	0.88	0.58	0.27
WSOC			1	0.71	0.22	0.70	0.51	0.44	0.74	0.48	0.78	0.86	0.64
EC				1	0.13	0.83	0.41	0.38	0.46	0.60	0.83	0.56	0.22
Ca^2+^					1	0.27	0.70	0.89	0.26	0.51	0.36	−0.16	−0.23
K^+^						1	0.67	0.51	0.51	0.69	0.96	0.59	0.34
Na^+^							1	0.80	0.14	0.55	0.70	0.18	0.14
Mg^2+^								1	0.05	0.73	0.58	0.16	−0.01
NH_4_^+^									1	0.22	0.48	0.96	0.94
F^−^										1	0.74	0.34	0.03
Cl^−^											1	0.55	0.30
NO_3_^−^												1	0.85
SO_4_^2−^													1

Notes: The number of PM_2.5_ samples was 45. PM_2.5_, fine particulate matter; OC, organic carbon; EC, elemental carbon; WSOC, water-soluble organic carbon.

## Data Availability

The data reported from this study is contained within the article (where applicable).
